# An analysis of material flow cost accounting in companies using different cost accounting systems

**DOI:** 10.1016/j.heliyon.2025.e42555

**Published:** 2025-02-10

**Authors:** Hayrettin Usul, Emre Betul Olgun

**Affiliations:** a Izmir Katip Celebi University, Institute of Social Sciences, Izmir, Turkiye; b Ministry of Finance, Tax Inspection Board, Izmir, Turkiye

**Keywords:** Material flow cost accounting, Case study, Job order costing, Process costing, Turkiye

## Abstract

Material Flow Cost Accounting[MFCA] is a tool of environmental management accounting that anticipates the quantitative and monetary tracking of waste generated in production processes. Reporting waste in quantitative terms holds significance in emphasizing its environmental impacts while calculating the cost of waste is crucial in revealing the cost incurred by waste generation for production companies. This study aims to analyse MFCA from the perspective of companies operating in the same region but utilizing different cost accounting systems. By examining two cases side by side, it is aimed to identify similarities and differences in practices. This paper further investigates potential challenges in implementing MFCA through an analysis of two companies: one employing a process costing system and the other employing a job order costing system. The first case study was conducted in a company engaged in powder coating production using a job order costing system, while the second case study was carried out in a company engaged in sunflower oil production employing a process costing system. MFCA was effectively implemented in both companies, revealing the cost of waste, additional costs incurred due to waste recycling, and the potential for savings. The results of Case Study 1 showed that the costs of products and material losses are 92.90 % and 7.10 %, respectively. The Case Study 1 also revealed that MFCA can be dynamically applied within the job order costing system during the production period due to the system's ability to directly track material and other resource usage, enabling detailed efficiency analyses for each individual order. The results of Case Study 2 indicated that products in QC(Quantity Center) 1 are 99.80 % of total outputs. However, this ratio includes by-products, which is 55.1 % of total outputs. In QC2, products account for 90.77 % of total outputs, while 9.23 % of them are material losses. The Case Study 2 revealed the shortcomings of MFCA in evaluating by-products.

## 1 Introduction

As a result of the increasing awareness accompanying global warming, environmental policies are now on the agendas of not only governments but also many corporations, especially multinational companies. The idea of reporting the negative impacts on the environment, along with the concept of sustainable production, has become a matter of public concern. Thus, production policies, including eco-efficiency policies that aim for a lower carbon footprint and are more environmentally conscious, are being targeted. These policies implemented in companies reduce input costs as well as costs related to waste. Therefore, transitioning to cleaner technologies as an extension of eco-efficiency policies offers both environmental and economic benefits [25,40,10]. Management instruments used to achieve sustainability goals have been developed during this process. Environmental management accounting, which can be considered one of these instruments, is utilized to merge companies' environmental objectives with their economic goals [24].

Environmental management accounting is a broad concept that encompasses many tools to achieve environmental and economic goals. One of these tools, Material Flow Cost Accounting [MFCA], is a branch of environmental management accounting that aims to track the monetary and quantitative aspects of waste generated, particularly as a result of production activities. MFCA emerges as one of the sole environmental management accounting tools that simultaneously reduces both environmental impact and cost of production [40]. Developed in Germany in the 1990s, this method started to be widely used in Japan [50]. MFCA is standardized through ISO 14051 in 2011.

MFCA is built upon two former methods based on cost flow. One of these is waste cost accounting, and the other is flow cost accounting. Waste accounting identifies environmental costs that can be prevented by eliminating waste. Flow cost accounting focuses on the relationship between corporate activities and environmental impacts, determining the economic and environmental development capacity related to processes and products [37].

MFCA is based on material flow analysis, an approach that tracks the flows and stocks of materials within a system by comparing all inputs and outputs to maintain a material balance Material balance, sometimes referred to as ‘input-output balance,’ ‘mass balance,’ ‘material flow balance,’ or ‘eco-balance,’ involves tracking all energy, water, materials, and wastes flowing into and out of a company. The physical mass balance of a company should ideally equate to zero [25].

MFCA has been developed to enhance resource efficiency in institutions, evaluate material flows within organizations, and make ecologically efficient decisions to improve both the economic and environmental performance of institutions [6,30]. There are numerous instruments within environmental management accounting available for examining the effects that production processes have on the environment. These tools can be used in conjunction with MFCA, or individually. Life Cycle Assessment is one of these instruments; it assesses the possible effects on the environment that are produced by a product's inputs, outputs, and life cycle. Life cycle costing is an additional technique that systematically evaluates the economic aspects of life cycle costs. These two tools are often used separately. The integration of these tools can benefit from the fundamental principles and methods of MFCA. MFCA evaluates the entire production system as input-output flows, categorizing these flows as material, energy, waste, and system costs [3]. Another prominent approach in environmental management accounting is the Sustainability Balanced Scorecard. Developed as an extension of the classical balanced scorecard to observe the increase in the company's performance from financial, customer, internal, and organization learning perspectives and an additional non-market perspective. Gathering data for creating the Sustainability Balanced Scorecard particularly emphasizes identifying energy and material flows [14]. As an effective tool in environmental management accounting, MFCA can be utilized in the process of creating the Sustainability Balanced Scorecard to report energy and material flows and reveal inefficient points.

MFCA tracks the resources used in the production process quantitatively and determines the amount of waste generated. In addition to the quantities of waste and products generated at each stage of production, the monetary values of these outputs are also identified. Thus, the costs associated with waste, which are typically included in the cost of product in traditional cost accounting, are additionally calculated.

Although MFCA calculates the cost of waste and products, it is primarily an instrument of environmental management accounting and is not a substitute for traditional cost accounting. However, it utilizes the same sources of information as traditional cost accounting. Unlike traditional cost accounting, it classifies costs differently, uses the term “quantity centers” instead of cost distribution centers, and introduces new concepts such as the material flow cost matrix that are not present in the traditional system [6,9,16,25,31]. Since MFCA is more of an environmental management tool than financial accounting, the expected economic success captured with MFCA is increased efficiency [34].

The information regarding the costs of waste obtained as a result of implementing MFCA is used as part of management accounting to increase efficiency. Due to increased efficiency, reductions are made in material waste, leading to a simultaneous decrease in the environmental impact of the production process, production costs, and the amount of energy used, consequently causing a decrease in carbon emission. Therefore, MFCA not only serves as a tool for cost control but also emerges as a tool used to control environmental impacts and achieve economic and environmental goals [32,15].

Most of the studies conducted so far have focused on the increased efficiency of MFCA in companies. The relationship between MFCA and traditional cost accounting has been theoretically examined in the literature. However, there has been no event-based study on the effectiveness of MFCA in terms of cost accounting systems. This study attempts to address this gap in the literature by analysing MFCA in conjunction with the cost accounting system used, through a case-based approach. It aims to identify differences in practices and potential challenges in implementing MFCA under different costing methods. Two separate companies operating in the same region in Turkiye have been selected to conduct two actual case studies. One of the companies involved in the study is a powder coating factory that employs a job order costing system, while the other is a sunflower oil factory that utilizes a process costing system.

This paper starts with a literature review of MFCA and a brief comparison with traditional cost accounting in Section 2. In Section 3, methodology and materials are explained. In Sections 4, 5 implementations of MFCA in the powder coating factory, followed by its application in the sunflower oil factory, are presented, respectively. The results of the case studies are discussed comparatively in Section 6. Finally, conclusions and future areas of research are addressed in Section 7.

## 2 MFCA

### 2.1 Literature review

MFCA has been developed to assess material flows within institutions, make ecologically efficient decisions, and enhance resource efficiency to improve the economic and environmental performance of organizations. Therefore, MFCA serves as a tool for ecological cost accounting, conducting cost calculations from an engineering perspective [6,16] As defined by The International Organisation for Standardisation in ISO 14051, MFCA is a tool for quantifying the flows and stocks of materials in processes or production lines in both physical and monetary units [22]. Since the publication of the ISO 14051 standard, various studies related to MFCA have been conducted in the literature.

It has been largely argued in the literature that MFCA increases efficiency by making waste visible. MFCA can be seen as one of the distinct environmental management accounting tools that simultaneously reduce environmental impacts and costs [40]. By tracking material flows throughout the production process, MFCA helps identify inefficiencies and quantifies the costs associated with non-product outputs, enhancing the transparency of environmental costs. This allows managers to explore various options for cost reduction [10]. The data on material waste costs obtained through MFCA can be integrated into management accounting to boost efficiency and decrease material waste, energy consumption, and carbon emissions [[32], [41]]. While accounting tools generally aim to balance sustainability with economic considerations, MFCA is particularly effective in reducing material waste, improving resource efficiency, and potentially lowering energy and water consumption, along with decreasing greenhouse gas emissions, making it more advantageous compared to other accounting tools [34].

MFCA is capable of being implemented not only within a single company but also integrated into various systems across value chains or geographical regions [47]. However majority of the studies on MFCA have focused on production performance in quantity centers with simple production structures. However, real-world production systems and quantity centers are much more complex in nature [9].

While some of the studies are in the form of reviews and discussions of MFCA[e.g.6,^15^, ^37^,^41^, ^50^], a significant portion of the studies conducted on MFCA have been in the form of case studies, typically carried out within a single company. Case studies have been conducted in various businesses such as the textile industry [7,[26], [42]], wood industry [5], beer production [13], starch production [23], color filter factory [20], oil refining [29], steel pipe manufacturing [38], turbine blade production [17], steel production [21], latex production [11], automotive parts manufacturing [46], hospitality [35] and paper production [10]. These case studies have examined MFCA from various perspectives. Thus far, these case studies have primarily observed increases in efficiency, waste management, and improved financial performance. Studies with a more general approach targeting specific sectors have also been conducted. For instance, in a study on the meat sector, it was found that MFCA identifies material, energy, and economic costs, identifying costs and quantities not included in traditional financial statements, thereby proving useful in decision-making processes [4]. Energy and cost analysis was conducted using MFCA in soybean production [8]Studies have also been conducted on the application of MFCA in the supply chain. A questionnaire study found that, to promote a low-carbon supply chain, the purchasing department must first understand the cost-cutting benefits of MFCA [32]. In another study, MFCA has been found to play a significant role in coordinating material flows within the supply chain and eliminating sub-optimization [18].

There are also studies that focus on the development of MFCA models, such as: the integration of Enterprise Resource Planning (ERP) and MFCA [12]; using MFCA in a supply chain collaboration model for life cycle assessment [33]; integration of life cycle costing and life cycle assessment using MFCA [3]; combining MFCA with the design of experiments (DOE) [5]; integration of MFCA with greenhouse gas (GHG) calculations [43]; an MFCA-based approach for waste recovery [52]; and combining MFCA with the circular economy [54].

Besides various benefits MFCA provide, there are some challenges of adopting MFCA in companies. A primary challenge in implementing MFCA is the limited data available from current accounting systems, which makes accurate cost allocation difficult [49]. Although MFCA is an effective environmental management tool, it cannot be used in dynamic analyses and is only applicable to static analyses [48]. MFCA is a retrospective tool that focuses on short-term management [1].

### 2.2 MFCA and traditional cost accounting

The purpose of cost accounting is to generate information for use in economic decision-making. Traditional cost accounting classifies costs into three main groups: direct material, direct labour, and general overhead costs, and provides unit cost information to use in economic decisions such as pricing policies. In traditional cost accounting, the cost of each input is considered in the total production cost of the product. Therefore, the production cost related to waste generated during production is also included in the product's cost. The rationale behind this calculation is that waste is considered an inevitable part of production.

The concept of cost accounting is based on calculating the value of consumed materials or, more precisely, determining the marginal benefit derived from the consumption of resources. Value-based costing starts with cash-based costing but extends this concept with opportunity costs as foregone profits. Hence, costs are the consumption of quantities or values [16]. Although it bears the name ‘cost accounting,’ MFCA didn't originate within cost accounting but rather from environmental management. In this sense, it would not be wrong to consider it as a management control mechanism, as this control system establishes a link between accounting and management [50]. In this regard, MFCA, as an environmental management accounting tool, is not a substitute for traditional cost accounting but rather a supplementary tool for it. MFCA provides a more detailed presentation of cost accounting, complementing the shortcomings of traditional cost accounting by offering a more comprehensive breakdown.

The most prominent difference in MFCA lies in its treatment of costs associated with waste. While traditional cost accounting assigns material losses to the product, MFCA treats material losses as independent cost objects [44]. This approach requires tracking material flows throughout the production system. MFCA also relies on the concept of QC, where inputs and waste are quantified in both physical and monetary terms [6].

Environmental costs in traditional cost accounting are included in the overhead costs. Placing these costs among overhead costs essentially hides these costs from management, leading to erroneous estimations of such costs [25]. On the other hand, MFCA allocates costs between products and waste, which makes waste visible to decision-makers. MFCA classifies costs in three main categories material costs, energy costs, and systems costs. Similar to overhead costs in traditional cost accounting, system costs include all costs other than material costs and energy costs including labour costs and overhead costs.

While MFCA and traditional cost accounting often draw information from the same sources, they produce different outcomes due to structural differences. In traditional cost accounting, waste, a normal outcome of production, is included in the product cost, whereas in MFCA, wastes are reported as a separate output. The aim here is to emphasize the environmental damage of waste quantitatively and the net loss to the company financially [16]. Information obtained through MFCA can be utilized in traditional cost accounting calculations. Cost and price predictions, product planning, and profit-loss statements exceed the purpose of MFCA [31]. MFCA values material wastes as economic losses and offers opportunities for management to reduce these wastes and enhance efficiency. It is easily adaptable to company resource planning [20]. It is evident that while traditional cost accounting struggles in identifying material wastes and costs, MFCA benefits in the detection and classification of incurred losses [7]. MFCA increases transparency regarding material flows and the costs attributable to these flows by allocating costs to both voluntarily produced outputs and involuntarily generated waste-like outputs. Thus, it reveals inefficient points related to material and energy consumption as well as hidden costs [51].

In traditional cost accounting, determining whether the total cost is covered by the sales amount is crucial, neglecting a definite determination regarding whether the material exits in the final product or turns into waste. Current methods of traditional cost accounting are insufficient in providing information regarding waste costs, whereas MFCA rectifies this inadequacy by assigning quantitative information to material flows [27,28]. While, MFCA exposes waste costs ignored by traditional cost accounting, presenting them to managers and other stakeholders, it doesn't aim to substitute conventional cost accounting systems but rather aims to complement the existing accounting systems.

## 3 Materials and methods

### 3.1 Methodology

This study aims to examine MFCA within the framework of two companies utilizing different cost accounting systems. Through the analysis of MFCA in two companies, the goal is to identify similarities and differences in their practices. Furthermore, the paper examines the challenges of implementing MFCA under different costing methods. Accordingly, an explanatory case study is chosen as the method as it is deemed suitable for answering research questions and providing information regarding the system's effectiveness. The case study method is one of the widely accepted qualitative research methods, focusing on a singular situation within its actual context [53]. Explanatory case studies are utilized to explain observed accounting practices. The focal point is a specific event used to comprehend and elucidate the theoretical structure of the event [39]. According to Berry and Oatley [2], case studies are highly suitable for management accounting research. They facilitate the establishment of the theoretical structure of processes within companies, contributing to a better understanding.

Case studies aim to generalize from single cases. However, case study research can be conducted in a single or multiple cases [53]. According to Seidman [45], multiple-case studies can strengthen the generalizations expected from the study. Examining the effectiveness of MFCA among companies employing different systems and cost allocation methods enhances the research's effectiveness. This study employs a multi-case studies approach to explore and examine differences between cases.

### 3.2 Selection of case companies

The purpose of this study is to analyse MFCA among companies operating in different sectors, thus having different production processes and utilizing different cost accounting systems. Therefore, two different companies in different sectors in the same region were initially selected. Among the selection criteria is that the companies where the case study will be conducted have different production processes and, consequently, different cost accounting systems.

The first case study was conducted in a powder coating manufacturing company in Turkiye for the year 2018. Production is carried out based on orders, and a separate production is conducted for each order. Therefore, the company applies the job costing method. On the other hand, the majority of production is carried out on a single production line.

The second case study was conducted in a sunflower oil manufacturing company in Turkiye for the year 2020. Production is carried out in different steps. Unlike the first case study methods, this company employs cost centers and a process costing system.

### 3.3 Data collection

As collecting and interviewing are among the methods used to collect data for case studies [53] both of these methods are used as data collection methods for the study. Financial and cost accounting information is collected from the accounting system of the companies. As stated in ISO 14051 management support is required to effectively implement MFCA, in-depth interviews with accounting managers, production managers, and other staff are used to understand the companies’ existing production process, cost structure, and accounting systems. Although all necessary information is obtained from companies, both companies' officials have insisted on keeping their company names confidential. All monetary data is presented in Turkish Lira[TL] and all quantitative data is presented in kilogram[kg].

### 3.4 Implementation of MFCA

This study aims to analyse MFCA in two case studies with two production systems and waste generation. Especially, MFCA has been aimed to be analyzed in terms of job order costing and process costing systems. Moreover, the types of waste produced by these two companies, recycling systems, and by-products generated during production differ. Case study 1 has a production system with internal recycling systems and no by-products whereas case study 2 has a production system with both internal and external recycling and by-products. So, the effectiveness of MFCA has been discussed in terms of these different situations. This study followed the implementation steps of MFCA outlined in clause 6 of ISO 14051. This includes the following steps.

PLAN: Involvement of management, determination of necessary expertise, specification of a boundary and a time period, determination of quantity centers.

DO: Identification of Inputs and outputs for each quantity center, quantification of the material flows in physical units, and quantification of the material flows in monetary units.

CHECK: Communication of MFCA results, MFCA data summary, and interpretation.

ACT: Identification and assessment of improvement opportunities.

## 4 Case study 1:Powder coating manufacturing company and MFCA

### 4.1 General information about the company

A powder coating factory in Turkiye, which applies the job order costing system in cost accounting, was selected for the first case study. The main product of the factory is powder coating for various industries including white goods, kitchen appliances, etc. Due to the specific characteristics of the customers’ orders, a unique production formula is prepared for each order, and the costs specific to this order are tracked.

### 4.2 Involvement of management and specification of boundaries and time period

During the meeting with the company management, MFCA is introduced. The accounting manager, production supervisors, and other personnel conducted discussions where the company's production line was examined and cost centers were planned. As a result of meetings with management, it has been decided that the timeframe for the study would be in 2018, and the study would focus on a product group that constitutes approximately 20 % of the production.

### 4.3 Determination of quantity centers and manufacturing process

The production process of powder coating begins with the withdrawal of raw materials from the warehouse and continues with mixing, extrusion, grinding, and crushing, followed by sieving, ending with packaging. Pigments, additives, and fillers along with resin and hardener are used in the manufacturing process. Besides these substances, auxiliary materials and packaging materials are also used.

**Fig. 1:**
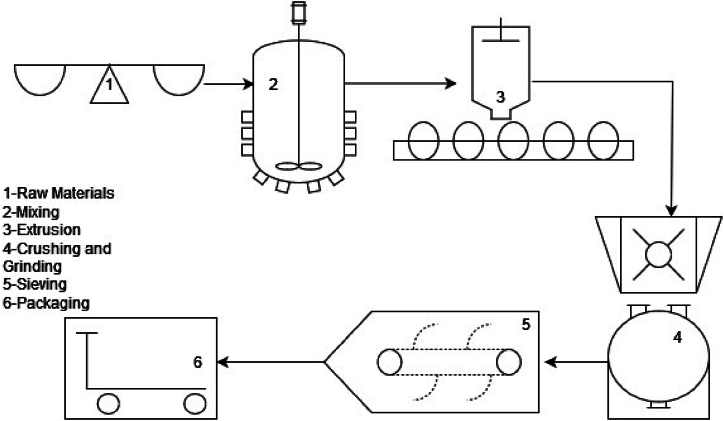
Powder coating manufacturing process.

Fig. 1 shows the manufacturing process and material flow. After weighing raw materials for production in step 1, the next step is premixing during which raw materials are blended to produce a homogenous mixture. In step 3(extrusion) the raw materials are crushed and mixed at a specific temperature in a machine called an extruder. The molten materials pass between cooling cylinders, forming layers of a certain thickness. These layers are subsequently divided into pieces in preparation for the step 4(crushing and grinding). The mixture previously turned into chips in the step 3 is ground at the crushing and grinding step. Since particles are expected to be of a specific standardized size larger particles are sifted out at step 5(sieving) and reintroduced into the production process. In step 6 (classifying and packaging), any oversize particles, which may have passed through the mill are eliminated to achieve a finely tuned particle size. The final product is then packaged for the order. There are three levels of the recovery system, which collect dust and recycle it internally to use as raw products. These recovery systems are filters, sieves, and cyclones.

In the existing production system, the quantification of materials is carried out at the beginning of production and during the packaging stage, hence there is only one quantity centre [QC 1] described in Table 1.

**Table 1:** Quantity centers.

Quantity Centre	Process Description
QC1	Raw materials are mixed, extruded, crushed, ground, sieved, and packaged in one single production line.

### 4.4 Identification and quantification of inputs and outputs in physical units

The main material is a special blend of resin, hardener, filler, pigments, and additives. Three types of outputs are generated during the manufacturing process. These are the final product, recycled waste, and disposed waste. Recycled waste is leftover material collected in the filters. Recycled waste is stored for use in subsequent orders. Disposed waste consists of losses generated during production, usually dispersed into the air. Since there is only one quantity center, mass balance is calculated based on the quantities at the beginning and the end of production. Table 2 shows the calculation of material balance.

**Table 2:** Material Balance-QC1.

INPUTS[kg]		OUTPUTS[kg]	
**Raw Materials**	313,045.13	**Product**	290,820.00
		**Recycled Waste**	12,292.00
		**Disposed Waste**	9933.13
**TOTAL**	313,045.13	**TOTAL**	313,045.13

### 4.5 Quantification of the material flows in monetary units

MFCA was conducted based on a specific product manufactured during the year 2018. The company applies job order costing systems and records manufacturing costs separately for each order. There are 165 separate orders regarding the specific product. Based on these orders, total costs and expenses are classified according to MFCA. Material costs include costs of raw materials, auxiliary materials, and packaging materials. System costs include all costs other than material costs and energy costs. The manufacturing costs of the specified product are shown in Table 3.

**Table 3:** Manufacturing Costs[TL].

**Material Costs**	3.287.469,37
**System Costs**	824.593,80
**Energy**	48.904,48
**TOTAL**	**4.160.967,65**

All costs summarized in Table 3, are allocated based on materials distribution percentage among outputs. The material costs, energy costs, and system costs are allocated based on the quantity of materials present in the positive and negative outputs. Material not existing in the final product and not collected in the filters is considered waste. Dust collected in the filters is considered recycled waste.

The material distribution ratio has been calculated as 92.90 % for products and 7.1 % for waste. These ratios have been determined by dividing the quantity of final products and the quantity of waste in Table 2 by the total material input, respectively.

### 4.6 MFCA data summary

The materials flow cost matrix in Table 4 shows the results of the allocation of materials, energy, and systems to products and wastes based on the materials distribution percentage. In terms of MFCA, outputs are classified as either products or waste. Although some of the waste is recycled internally, all the output other than finished products including recycled waste is considered waste.

**Table 4:** Material flow cost matrix.

	Quantity [kg]	Material [TL]	Energy [TL]	System [TL]	Total [TL]
Total Input	313,045.13	3,287,469.37	48,904.48	824,593.80	4,160,967.65
Product[92.90 %]	290,820.00	3,054,070.29	45,432.43	766,050.46	3,865,553.19
Material Losses[7.1 %]	22,225.13	233,399.07	3472.05	58,543.34	295,414.46
*Recycled Waste[3.93 %]*	*12,292.00*	*129,085.46*	*1920.28*	*32,378.42*	*163,384.15*
*Other Waste[3.17 %]*	*9933.13*	*104,313.62*	*1551.77*	*26,164.92*	*132,030.31*
Total Output	313,045.13	3,287,469.37	48,904.48	824,593.80	4,160,967.65

### 4.7 Findings based on an analysis of the material flow cost accounting implementation and improvement opportunities

92.90 %, of the inputs involved in the production process, have been transformed into the final product, while the remaining 7.1 % have become material losses Recyclable waste accounts for 3.93 % of the total output. These recyclable wastes contribute to production cost savings but have some drawbacks such as storage costs and waiting time until the next production order.

Moreover, the energy and system costs incurred to produce these wastes create an additional burden for the company. The additional cost amounts to 34,298.70 TL, which will be transferred as an extra cost to the subsequent production process. On the other hand, the quantity of non-recyclable waste accounts for 3.17 % of the total output, which amounts to 9.933,13 kg. In other words, approximately 9 tons of waste, in the form of air, water, or solid waste, are released into the environment. Besides the greenhouse gas emissions caused by these wastes, the cost incurred during the production process for these wastes is 132,030.31 TL. For MFCA, this amount represents the sole target indicating the company's potential savings. Since, reducing the amount of waste will lead to a further decrease in the cost of production, reporting the cost of waste separately will serve as an indicator for company managers to establish performance goals.

On the other hand, while the process of incorporating dust products accumulated in the filtration mechanisms at the facility into production represents a form of internal recycling for MFCA the aim is to minimize or eliminate waste, even if recyclable. Internal recycling within the company incurs new costs in each production process. The amount of material losses collected in cyclones, filters, and screens is 12,292 kg. These wastes are collected for the production of the next order of the same product and are reintegrated into the production process. However, besides material expenses for producing these wastes, there are energy and system costs incurred. The total cost incurred amounts to 163,384.15 TL. Out of this cost related to recyclable waste, 1920.28 TL is from energy expenses, and 32,378.42 TL is from system expenses.

Although these wastes included as raw materials in the next order of production do not create material expenses in the subsequent production stage, they are included in production with energy and system costs amounting to 34,298.70 TL. In the next order, they continue to generate new production costs. Additionally, they create greenhouse gas emissions again in the subsequent production stage. Therefore, besides the financial burden on the company, the environmental damage they cause also accumulates. Hence, MFCA's goal, even for recyclable waste, is to minimize all waste to the lowest possible level, achieving both economic and environmental performance.

## 5 Case study 2: sunflower oil manufacturing

### 5.1 General information about the company

A sunflower oil manufacturing company in Turkiye that applies a process costing system was selected for the second case study. The company manufactures sunflower oil in three facilities in the factory. These three facilities also serve as cost centers for process costing systems. The main ingredient is sunflower seeds purchased from local farmers.

### 5.2 Involvement of management and specification of boundaries and time period

During the meeting with the accounting manager, production supervisors, and other personnel, MFCA was introduced. During discussions and observation of the manufacturing process, the company's production line was examined and quantity centers were planned. As a result of meetings with management, it was decided that the timeframe for the study would be in 2020, and the study wouldn't have a boundary in terms of product.

### 5.3 Determination of quantity centers and manufacturing process

The sunflower oil factory has a production system consisting of three main stages carried out in three production facilities. These stages are as follows:

● Oil Pressing and Extracting
● Refinement
● Filling and Packaging.

Oil pressing and extracting: During oil pressing and extracting, crude oil and oil cakes are produced out of sunflower seeds. This step includes cleaning and dehulling of seeds, grinding and oil extraction, crude oil extraction with solvents, and removal of solvents. The main inputs are seeds, water, and solvents, the main outputs are crude oil and oil cake. Wastes generated in this process consist of wastewater, dust, and solvent.

Refinement: The refining process is a set of procedures applied to crude oil to remove all unwanted substances from the oil to obtain clear oil. These procedures include neutralizing, bleaching, degumming, and deodorizing. The main inputs of this process are crude oil and auxiliary material and the main output is refined oil. Soap stock, spent earth and acid oil are by-products of the process.

**Fig. 2:**
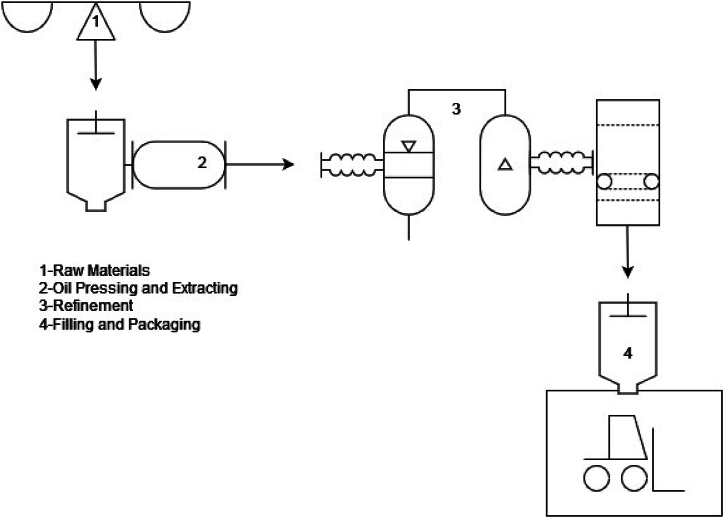
Sun flower oil manufacturing process.

Filling and packaging: During the filling and packaging process, refined oil is filled into plastic bottles and tin cans, and subsequently placed as packaged sunflower oil into boxes and pallets. No wastage is recorded at the filling and packaging facility. Fig. 2 summarizes the manufacturing process.

Sunflower oil manufacturing is carried out in three steps across three facilities. Input-output measurements are conducted at each production facility. From an MFCA perspective, quantity centers can be established at every stage where input-output balance can be assured. Therefore, the crude oil production, refining, and filling-packaging facilities, where the main stages of production take place, have been designated as quantity centers for MFCA. Table 5 shows the quantity centers determined for MFCA implementation.

**Table 5:** Quantity centers.

Quantity Centre	Process Description
QC1	Sunflower seeds are pressed and crude oil and oil cakes are produced
QC2	As a result of the refinement of crude oil, refined oil is produced
QC3	Refined oil is packaged.

The company applies process costing to calculate the unit cost of sunflower oil. Total costs and expenses classified based on MFCA principles are shown in Table 6.

**Table 6:** Manufacturing costs.

	QC1	QC2	QC3
**Material Costs[TL]**	74,751,581.11	425,598,102.74	435,445,827.83
**Energy Costs[TL]**	1,227,202.81	3,988,642.45	182,590.81
**System Costs[TL]**	2,052,035.41	5,377,328.49	6,884,162.25
**Total Costs[TL]**	78,030,819.33	434,964,073.68	442,512,580.89

### 5.4 Identification and quantification of inputs and outputs in physical units

Material balance has been established at each of the three QCs of the factory. Inputs in QC2 include outputs of QC1[crude oil] as well as beginning inventory. Similarly, inputs in QC3 include the output of QC2[refined oil] Table 7 summarizes the input-output balance of each of QCs.

**Table 7:** Material balance.

	Inputs[kg]	Outputs[kg]
**QC1**		
Seed	23,975,762.00	
Hexan	49,390.00	
Steam	35,160.00	
Crude Oil		10,735,331.00
Oil Cake		13,275,591.00
Hexan[recy.]		47,384.77
Hexan Waste		2005.23
**TOTAL**	**24,060,312.00**	**24,060,312.00**
**QC2**		
Crude Oil	70,345,210.00	
*From 1. step*	*[10,735,331*.*00]*	
*Beginning inventory*	*[59,609,879*.*00]*	
Auxiliary Materials	2,889,746.91	
Refined Oil		66,491,229.00
Spent Earth		467,010.00
Soap Stock		5,330,019.00
Acid Oil		95,151.00
Waste		851,548.00
**TOTAL**	**73,234,957.00**	**73,234,957.00**
**QC3**		
Refined Oil	65,179,485.00	
Packaged Oil		65,179,485.00
**TOTAL**	**65,179,485.00**	**65,179,485.00**

### 5.5 Quantification of the material flows in monetary units

Manufacturing costs specified in Table 6 are allocated to products and material losses based on materials distribution centers. However, the production process includes by-products and recycled materials. So each quantity centre has different calculation methods to allocate costs. Table 8 shows the allocation of costs among outputs based on the physical units of materials presented in Table 7.

**Table 8:** 

Quantity Centre	Outputs	Allocation Key	Distribution Percentage
**QC1**	Crude Oil[X_1_]	$\frac{X_{1}}{\sum_{i=1}^{2}X_{i}}$	45.00 %
	Oil Cake[X_2_]	$\frac{X_{2}}{\sum_{i=1}^{2}X_{i}}$	55.00 %
	Hexane[Y_1_]	$\frac{Y_{1}}{\sum_{i=1}^{2}Y_{i}}$	95.04 %
	Hexane Waste[Y_1_]	$\frac{Y_{2}}{\sum_{i=1}^{2}Y_{i}}$	4.06 %
**QC2**	Refined Oil[Z_1_]	$\frac{Z_{1}}{\sum_{i=1}^{5}Z_{i}}$	90.79 %
	Spent Earth[Z_2_]	$\frac{Z_{2}}{\sum_{i=1}^{5}Z_{i}}$	0.64 %
	Soap Stock[Z_3_]	$\frac{Z_{3}}{\sum_{i=1}^{5}Z_{i}}$	7.28 %
	Acid Oil[Z_4_]	$\frac{Z_{4}}{\sum_{i=1}^{5}Z_{i}}$	0.13 %
	Waste[Z_5_]	$\frac{Z_{5}}{\sum_{i=1}^{5}Z_{i}}$	1.16 %
**QC3**	Packaged Oil[P_1_]	$\frac{P_{1}}{\sum_{i=1}^{1}P_{i}}$	100.00 %

**Fig. 3:**
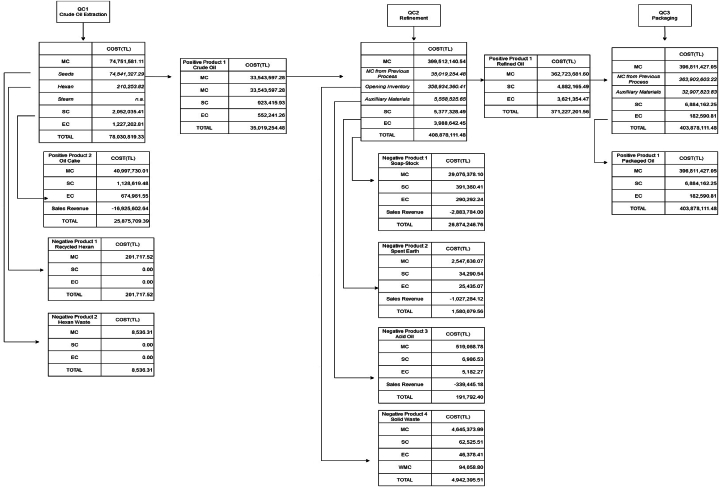
MFCA cost allocation.

The positive product from the previous quantity center is included as an input into the subsequent quantity center. A portion of the positive product produced in the first quantity center has been transferred to the second quantity center. Additionally, the crude oil in the beginning inventory of the second quantity center has been included as an input in production. Similarly, a portion of the positive product in the second quantity center has been transferred to the third quantity center, transforming it into the final product. Across all quantity centers, the material costs of both positive and negative products have been allocated considering the quantities of materials present in these outputs. 95.94 % of the hexane gas included in production at the first quantity center is recycled and reintroduced into production. The cost of the recycled hexane gas and the hexane gas that becomes waste among the outputs of the first quantity center consists solely of material costs. Apart from this, the system and energy costs allocated to all other outputs have been calculated based on the quantity of materials in these outputs. The MFCA cost allocation is illustrated in Fig. 3. The first QC contains four different outputs. Among these, crude oil has been regarded as a positive product. Oil cake, considered a by-product from the perspective of classical cost accounting, has been accepted as a second positive product due to both its proportion in the total output quantity and its market value. The steam used in QC1 increases the moisture of the oil cake, thereby contributing to the weight of the oil cake. According to ISO 14051, by-products can be reported as either positive or negative outputs. Furthermore, according to this standard, all wastes, whether recycled or not, are considered negative outputs. Therefore, hexane gas utilized in production through recycling and hexane gas lost as waste are reported separately as negative outputs. In the second quantity center, among the five different outputs, refined oil is a positive product, while soap stock, spent earth, acid oil, and solid waste are negative outputs. Spent earth is recycled by recovering the oil from the bleaching earth. Soap stock is utilized in soap manufacturing, while acid oil, rich in fatty acids, serves as an input in various industries. Due to these properties, soap stock, spent earth, and acid oil all hold market value. Their sales revenues were subtracted from the total costs to find their net costs. Waste management costs related to solid waste have been added to its cost. At the third quantity center, no waste has been reported, and all inputs have been transformed into positive products.

### 5.6 MFCA data summary

Positive and negative product costs together with their proportions are summarized in the material flow cost matrix in Table 9.^1^ The waste costs include the costs of hexane emissions and recyclable hexane at the first QC, as well as the costs related to spent earth, soap stock, acid oil, and solid waste generated at the second QC. Since steam expenses are recorded in general overhead costs, they are included in system costs. The income obtained from the sale of waste is not included in the table. Similarly, the sales revenues related to oil cake produced as a by-product at the first QC and considered a positive output within the scope of this study, are also not included.

**Table 9:**
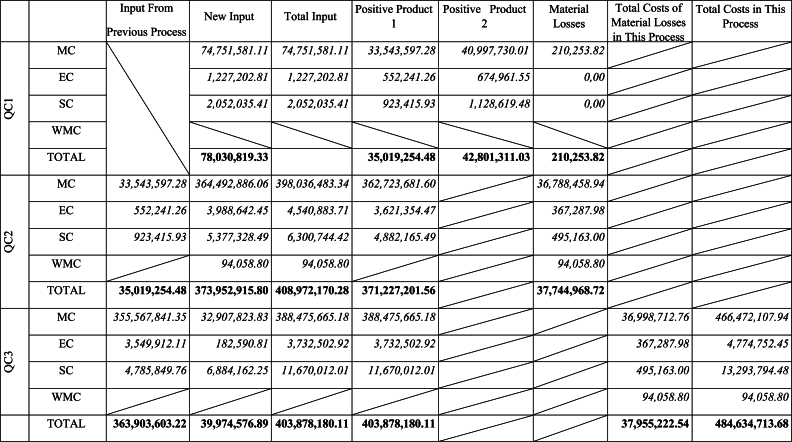
Material flow cost Matrix.

### 5.7 Findings based on an analysis of the material flow cost accounting implementation

The main outputs of the first QC are crude oil and oil cake. Products in QC 1 are 99.80 % of total outputs. However, this ratio includes by-products which is 55.1 % of total outputs. Oil cake is considered a by-product of traditional cost accounting. Due to its market value, it was sold in the relevant year. In this study, oil cake has been considered as a positive product. According to ISO 14051, by-products can be categorized as material waste or positive products based on the nature of the process. The oil cake obtained at the first QC is sold for use as animal feed. Even though the intended output of production is crude oil, the oil cake produced as a significant by-product, with considerable market value, constitutes 55 % of the total output. While the cost of oil cake is not calculated from the perspective of traditional cost accounting, it is calculated for Material Flow Cost Accounting [MFCA]. The crucial point here is whether the oil cake, constituting 55 % of the outputs in terms of mass proportion, will be considered a positive or negative product in terms of MFCA. ISO 14051 has provided flexibility in this regard. Although there are findings in the literature regarding the calculation of costs for by-products in MFCA, there isn't a clear consensus on reporting by-products as waste or positive products. Guenther et al. [16] mentioned that MFCA categorizes unwanted outputs, such as by-products, as compound products and allocates costs based on their physical quantities. Ho et al. [19] stated that MFCA evaluates unwanted by-products and wastes similarly and assigns them a share of process costs. Nakajima [31] expressed that MFCA, unlike traditional cost accounting, focuses on the environmental aspect of productivity rather than whether costs are covered by sales. According to the author, while traditional cost accounting lacks the concept of cost for waste and other outputs generated during the production of the intended product, MFCA calculates the cost of both by-products and waste. In terms of MFCA, minimizing waste moreover zero waste is the final target. While some of the waste can be minimized by reducing the resources used or changing the production systems, some of the wastes or by-products can't be prevented. In this regard, oil cake is such an output that can't be prevented. It is a compulsory part of the production. From this perspective, reporting oil cake as waste can lead to misunderstandings in terms of corporate management [36]. Since it has a natural origin and is unpreventable, oil cake has been considered a second positive product. The most effective way to reduce oil cake is to procure sunflower seeds that are more efficient in terms of oil content. In this case, by expanding MFCA to the cultivation phase of sunflower seeds, the previous link in the supply chain, more efficient agricultural production in terms of oil content, can be achieved. The positive output obtained from production in the second QC is refined oil. Additionally, spent earth, soap stock, and acid oil, considered by-products by traditional cost accounting, along with solid waste have been classified as waste. The cost ratio of refined oil, a positive product at the second quantity center, to the total costs is 90.79 %. The greatest financial loss due to waste formation occurred at the second quantity center. The process where energy costs are highest is the second quantity center, while the process with the highest system costs is the third quantity center.

## 6 Discussions about the effectiveness of MFCA under different cost accounting systems

This study examines the effectiveness of MFCA under different cost accounting systems in two different case studies. Case Study 1 was conducted in a powder coating manufacturer using job order costing method.

The job order costing system is a cost system utilized by companies producing unique products, where direct expenses are tracked to the cost object. The cost object appears as each unique order or product produced upon request. Hence, the job order costing system calculates costs separately for each order or product. Unlike process costing, in the job order costing system, as each order uses different amounts of resources, the allocation of resources to each order on an average basis is not applicable. Therefore, in terms of material flows, it can be argued that the job order costing system provides more accurate results. Throughout a period, a separate order card and cost record are maintained for each order. In a specific product type production based on a total of 165 orders at a powder coating plant, costs reported under MFCA have been tracked on a per-order card basis.

MFCA is generally used as an end-of-period analysis tool. The analysis done at the end of the year sheds light on the next periods. Since MFCA cannot be utilized in dynamic analyses, it is only applicable in static analyses [48]. One reason for this is that MFCA reports the waste generated in the production process at the end of a period. Therefore, it provides company managers with the opportunity for retrospective analysis, while steps toward efficient production can be taken in subsequent periods.

Case Study 1 revealed that MFCA can be dynamically applied with a job order costing system and varying waste quantities have been observed for different orders of the same product recipe within the period. An example related to a specific product of powder coating is presented in Table 10. While the product recipe remains the same, the waste generated in production in November is higher compared to other months. With such information, a more detailed investigation can be conducted on orders with higher waste generation to identify micro-conditions causing these wastes, which can be addressed for future prevention. The study conducted in the powder coating plant revealed that MFCA when integrated into the job order costing system, can serve as a dynamic analytical tool.

**Table 10:** Job cost sheets of the specific product.

	30.03.2018	30.04.2018	11.05.2018	31.05.2018	30.11.2018	21.12.2018
Materials	9361.94	9110.70	7111.70	6720.10	6551.74	10,285.64
Total Waste	1587.71	1342.64	850.50	953.00	1111.12	1744.36
Recycled Waste	1274.26	860.46	454.36	560.01	509.58	971.42
Disposed Waste	313.44	482.18	396.14	392.94	601.54	772.94

Case Study 2 has been conducted in a sunflower oil manufacturer using process costing method. The process costing system calculates the total cost for all manufactured goods and then divides it by the total quantity of manufactured goods to find the unit cost. The most significant difference between process costing and job order costing systems lies in the utilization of average unit costs. In the process costing system, as production occurs in a continuous flow, the resources used are allocated to the total cost of the manufactured goods, followed by the calculation of unit costs. Both the sunflower seeds, inputs in sunflower oil production, and the refined oil, the output in oil production are homogeneous products that cannot be distinguished from one another. Sunflower oil production inherently necessitates the application of a process costing system. This is because the produced oil is collected in tanks, and the total cost is calculated collectively. In this production and costing system, MFCA can be used as an end-of-period analysis tool. However, the operational structure of the process costing system does not hinder the smooth implementation of MFCA.

MFCA establishes a link between accounting and management as a control mechanism, facilitating the identification of inefficient points. By accurately calculating the cost of waste in monetary terms, MFCA enhances the transparency of environmental costs, providing managers with opportunities to explore different options to reduce costs. Consequently, managers make more accurate investment decisions and can evaluate the benefits brought by cleaner production technologies [10,50]. Both financial accounting, cost accounting, and MFCA generate information from the same data flows within the company. While cost accounting information is used in pricing products and fundamental profitability analyses, it falls short in efficiency analysis and environmental performance. MFCA addresses this gap by simultaneously revealing the impact of waste on both the environment and the company's financial structure. As long as material flows are identified and waste quantities are determined, MFCA can be effectively utilized regardless of which conventional cost accounting tool is used in determining product costs.

## 7 Conclusions

This study examines the implementation of MFCA in two different case studies from various sectors with different cost accounting systems. MFCA focuses on calculating the cost of waste but cannot be regarded as a tool for classical cost accounting or a substitute for it. In a broader sense of environmental management accounting, MFCA serves as an instrument within management accounting. It operates as a method that aids decision-making and policy development tailored to company needs, supplementing the records and reports maintained in classical cost accounting and financial accounting. Both cost accounting, financial accounting, and MFCA benefit from material flow within the company. However, these three systems generate information for different purposes using the same data. Due to MFCA's tracking and cost calculation of waste, it possesses an inclusive structure that encompasses classical cost accounting.

When analyzed across different cost accounting systems, MFCA appears to maintain its effectiveness. Leveraging material flows and financial data, calculations made compatible with MFCA at the end of the period shed light on environmental performance and efficiency analysis beyond the promises of traditional cost accounting. As mentioned by Nakajima [31], An important characteristic of MFCA is its comprehensive nature that extends beyond traditional cost accounting. Both MFCA and cost accounting utilize the company's resource utilization as a primary source of information. This study reveals not the effectiveness of MFCA in different accounting systems, but rather how effective material flows are in different cost accounting systems. The job order costing system is a cost system used by firms producing unique products, where direct expenses are traced to the cost object. The cost object appears as each specific order or product produced upon request. On the other hand, process costing is applied by firms producing a large number of similar products. In this case, the cost object becomes the total products of the same kind produced. Hence, while the job order costing system calculates separate costs for each order or product, the process costing system computes the total cost for all produced goods, divides it by the total quantity of products, and finds the average unit cost.

If MFCA together with the job order costing system is used within the period rather than solely at period-end, efficiency analyses and environmental performance evaluations can be conducted for each order, surpassing the benefits of the end-of-period approach. This situation arises not solely from an additional benefit of MFCA but stems from the operational principle of the job order costing system. The process costing system, however, calculates costs based on average unit prices over stages, unlike the job order costing system that tracks flows on an order basis. MFCA can serve as an analysis tool within the process costing system at period-end and can also be used at specific intervals within the period.

The first case study revealed that while internal recycling within a company saves material costs, it leads to additional system and energy costs. The second case study highlighted the different outcomes arising from evaluating certain products considered as by-products in traditional cost accounting, either as waste or products in the context of MFCA. These results revealed certain drawbacks. When these by-products, with a high share in the total outputs, are reported as products in MFCA, it leads to misleading assessments because these products are not the ultimate goal of production, and their sales revenues are lower compared to the intended final main product. On the other hand, when these products are reported as waste in MFCA, their high share in the outputs and their higher economic value compared to other wastes lead to misleading evaluations [36]. Therefore, exploring how these by-products should be assessed in terms of MFCA can be considered a future area of research.

## CRediT authorship contribution statement

**Hayrettin Usul:** Writing – review & editing, Writing – original draft. **Emre Betul Olgun:** Writing – review & editing, Writing – original draft.

## Disclaimer

The manuscript was originally written in Turkish **without any assistance of AI Technologies**. Chat GPT was used in some parts of the manuscript just for **translation purposes**. The translated parts generated by Chat GPT were carefully controlled and adjusted.

## Data availability statement

Data will be made available on request.

## Declaration of competing interest

The authors declare that they have no known competing financial interests or personal relationships that could have appeared to influence the work reported in this paper.

## Footnotes

1 The material, energy, and system costs at the second quantity center were found by dividing the output quantity of 66,491,229 kg in that quantity center, resulting in unit costs of 5.46 TL, 0.05 TL, and 0.07 TL for material, energy, and system, respectively. These unit costs were then multiplied by the input quantity of 65,179,485 kg in the third quantity center to calculate the material, energy, and system costs transferred from the previous stage in the third quantity center.
